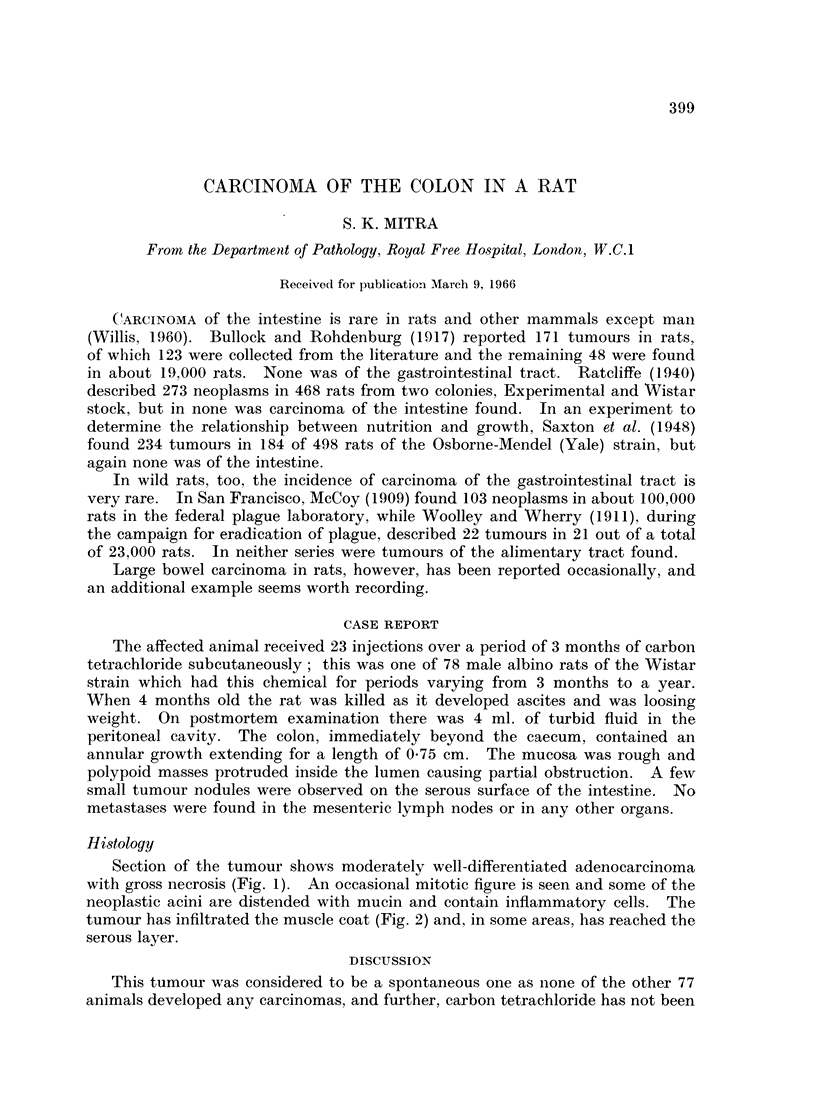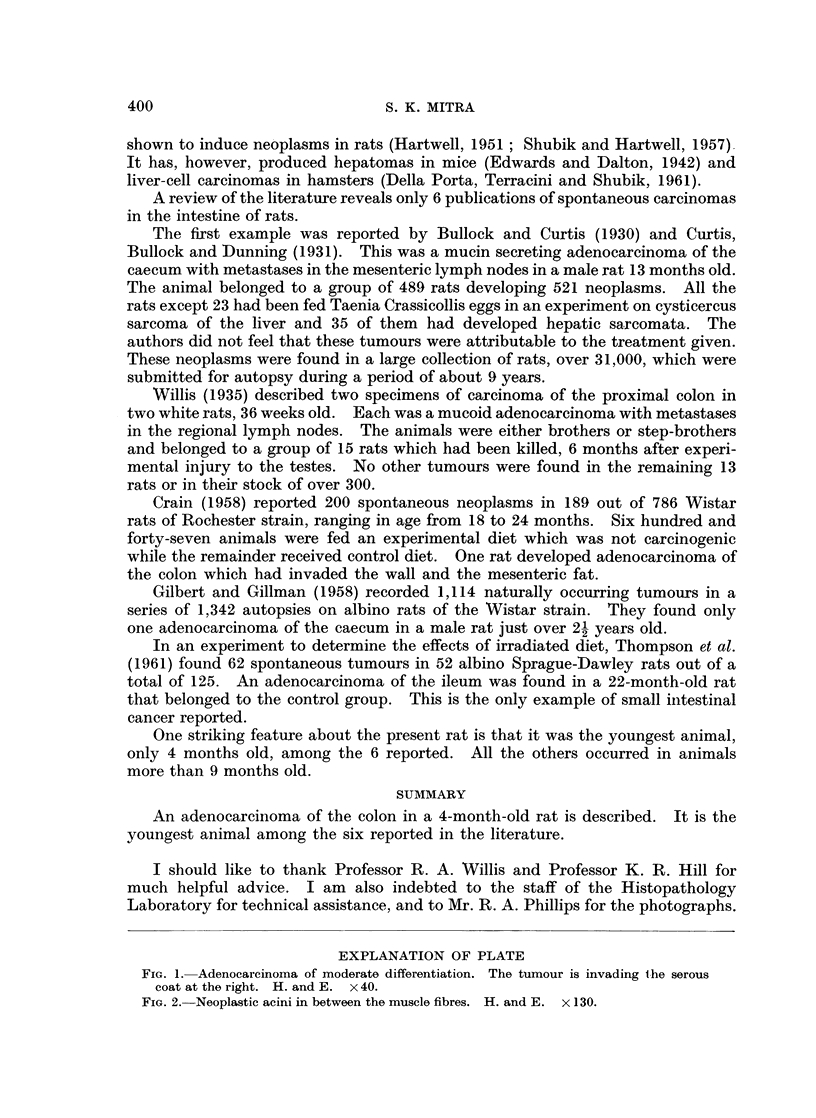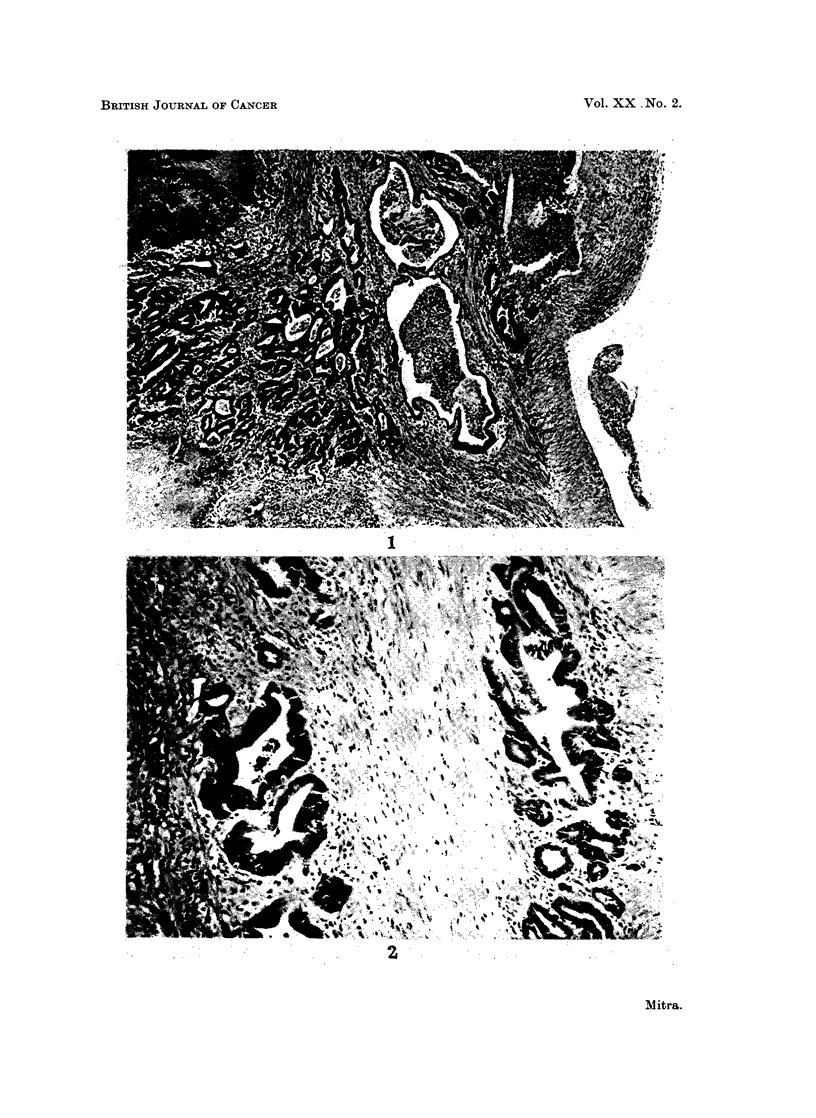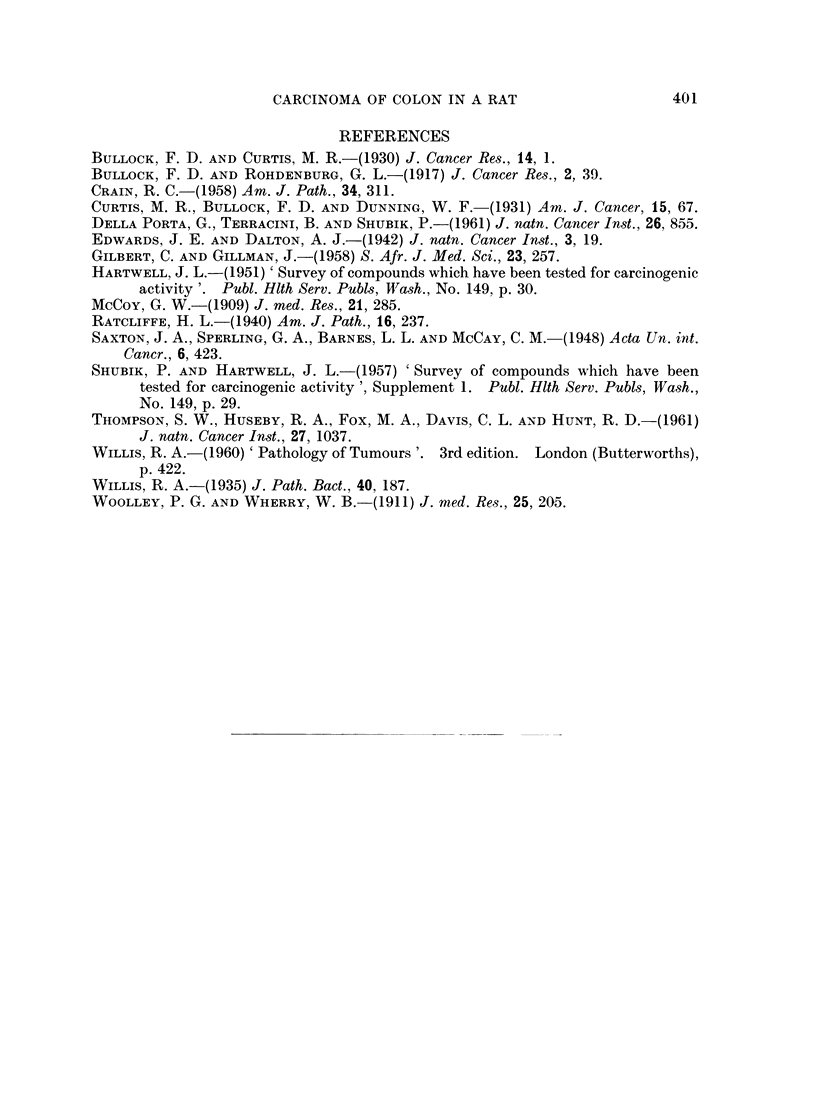# Carcinoma of the colon in a rat.

**DOI:** 10.1038/bjc.1966.47

**Published:** 1966-06

**Authors:** S. K. Mitra

## Abstract

**Images:**


### CARCINOMA OF THE COLON IN A RAT

S. K. MITRA

*From the Department of Pathology, Royal Free Hospital, London, W.C.*1

Received for publication March 9, 1966

CARCINOMA of the intestine is rare in rats and other mammals except man (Willis, 1960). Bullock and Rohdenburg (1917) reported 171 tumours in rats, of which 123 were collected from the literature and the remaining 48 were found in about 19,000 rats. None was of the gastrointestinal tract. Ratcliffe (1940) described 273 neoplasms in 468 rats from two colonies, Experimental and Wistar stock, but in none was carcinoma of the intestine found. In an experiment to determine the relationship between nutrition and growth, Saxton *et al.* (1948) found 234 tumours in 184 of 498 rats of the Osborne-Mendel (Yale) strain, but again none was of the intestine.

In wild rats, too, the incidence of carcinoma of the gastrointestinal tract is very rare. In San Francisco, McCoy (1909) found 103 neoplasms in about 100,000 rats in the federal plague laboratory, while Woolley and Wherry (1911), during the campaign for eradication of plague, described 22 tumours in 21 out of a total of 23,000 rats. In neither series were tumours of the alimentary tract found.

Large bowel carcinoma in rats, however, has been reported occasionally, and an additional example seems worth recording.

### CASE REPORT

The affected animal received 23 injections over a period of 3 months of carbon tetrachloride subcutaneously ; this was one of 78 male albino rats of the Wistar strain which had this chemical for periods varying from 3 months to a year. When 4 months old the rat was killed as it developed ascites and was loosing weight. On postmortem examination there was 4 ml. of turbid fluid in the peritoneal cavity. The colon, immediately beyond the caecum, contained an annular growth extending for a length of 0·75 cm. The mucosa was rough and polypoid masses protruded inside the lumen causing partial obstruction. A few small tumour nodules were observed on the serous surface of the intestine. No metastases were found in the mesenteric lymph nodes or in any other organs.

### *Histology*

Section of the tumour shows moderately well-differentiated adenocarcinoma with gross necrosis (Fig. 1). An occasional mitotic figure is seen and some of the neoplastic acini are distended with mucin and contain inflammatory cells. The tumour has infiltrated the muscle coat (Fig. 2) and, in some areas, has reached the serous layer.

### DISCUSSION

This tumour was considered to be a spontaneous one as none of the other 77 animals developed any carcinomas, and further, carbon tetrachloride has not been shown to induce neoplasms in rats (Hartwell, 1951 ; Shubik and Hartwell, 1957). It has, however, produced hepatomas in mice (Edwards and Dalton, 1942) and liver-cell carcinomas in hamsters (Della Porta, Terracini and Shubik, 1961).

A review of the literature reveals only 6 publications of spontaneous carcinomas in the intestine of rats.

The first example was reported by Bullock and Curtis (1930) and Curtis, Bullock and Dunning (1931). This was a mucin secreting adenocarcinoma of the caecum with metastases in the mesenteric lymph nodes in a male rat 13 months old. The animal belonged to a group of 489 rats developing 521 neoplasms. All the rats except 23 had been fed Taenia Crassicollis eggs in an experiment on cysticercus sarcoma of the liver and 35 of them had developed hepatic sarcomata. The authors did not feel that these tumours were attributable to the treatment given. These neoplasms were found in a large collection of rats, over 31,000, which were submitted for autopsy during a period of about 9 years.

Willis (1935) described two specimens of carcinoma of the proximal colon in two white rats, 36 weeks old. Each was a mucoid adenocarcinoma with metastases in the regional lymph nodes. The animals were either brothers or step-brothers and belonged to a group of 15 rats which had been killed, 6 months after experimental injury to the testes. No other tumours were found in the remaining 13 rats or in their stock of over 300.

Crain (1958) reported 200 spontaneous neoplasms in 189 out of 786 Wistar rats of Rochester strain, ranging in age from 18 to 24 months. Six hundred and forty-seven animals were fed an experimental diet which was not carcinogenic while the remainder received control diet. One rat developed adenocarcinoma of the colon which had invaded the wall and the mesenteric fat.

Gilbert and Gillman (1958) recorded 1,114 naturally occurring tumours in a series of 1,342 autopsies on albino rats of the Wistar strain. They found only one adenocarcinoma of the caecum in a male rat just over $2\frac{1}{2}$ years old.

In an experiment to determine the effects of irradiated diet, Thompson *et al.* (1961) found 62 spontaneous tumours in 52 albino Sprague-Dawley rats out of a total of 125. An adenocarcinoma of the ileum was found in a 22-month-old rat that belonged to the control group. This is the only example of small intestinal cancer reported.

One striking feature about the present rat is that it was the youngest animal, only 4 months old, among the 6 reported. All the others occurred in animals more than 9 months old.

### SUMMARY

An adenocarcinoma of the colon in a 4-month-old rat is described. It is the youngest animal among the six reported in the literature.

I should like to thank Professor R. A. Willis and Professor K. R. Hill for much helpful advice. I am also indebted to the staff of the Histopathology Laboratory for technical assistance, and to Mr. R. A. Phillips for the photographs.

---

### EXPLANATION OF PLATE

FIG. 1.—Adenocarcinoma of moderate differentiation. The tumour is invading the serous coat at the right. H. and E. ×40.

FIG. 2.—Neoplastic acini in between the muscle fibres. H. and E. ×130.

1

2

### REFERENCES

BULLOCK, F. D. AND CURTIS, M. R.—(1930) *J. Cancer Res.*, **14**, 1.
BULLOCK, F. D. AND ROHDENBURG, G. L.—(1917) *J. Cancer Res.*, **2**, 39.
CRAIN, R. C.—(1958) *Am. J. Path.*, **34**, 311.
CURTIS, M. R., BULLOCK, F. D. AND DUNNING, W. F.—(1931) *Am. J. Cancer*, **15**, 67.
DELLA PORTA, G., TERRACINI, B. AND SHUBIK, P.—(1961) *J. natn. Cancer Inst.*, **26**, 855.
EDWARDS, J. E. AND DALTON, A. J.—(1942) *J. natn. Cancer Inst.*, **3**, 19.
GILBERT, C. AND GILLMAN, J.—(1958) *S. Afr. J. Med. Sci.*, **23**, 257.
HARTWELL, J. L.—(1951) ‘ Survey of compounds which have been tested for carcinogenic activity ’. *Publ. Hlth Serv. Publs, Wash.*, No. 149, p. 30.
MCCOY, G. W.—(1909) *J. med. Res.*, **21**, 285.
RATCLIFFE, H. L.—(1940) *Am. J. Path.*, **16**, 237.
SAXTON, J. A., SPERLING, G. A., BARNES, L. L. AND MCCAY, C. M.—(1948) *Acta Un. int. Cancr.*, **6**, 423.
SHUBIK, P. AND HARTWELL, J. L.—(1957) ‘ Survey of compounds which have been tested for carcinogenic activity ’, Supplement 1. *Publ. Hlth Serv. Publs, Wash.*, No. 149, p. 29.
THOMPSON, S. W., HUSEBY, R. A., FOX, M. A., DAVIS, C. L. AND HUNT, R. D.—(1961) *J. natn. Cancer Inst.*, **27**, 1037.
WILLIS, R. A.—(1960) ‘ Pathology of Tumours ’. 3rd edition. London (Butterworths), p. 422.
WILLIS, R. A.—(1935) *J. Path. Bact.*, **40**, 187.
WOOLLEY, P. G. AND WHERRY, W. B.—(1911) *J. med. Res.*, **25**, 205.